# The safety and feasibility of a Halliwick style of aquatic physiotherapy for falls and balance dysfunction in people with Parkinson's Disease: A single blind pilot trial

**DOI:** 10.1371/journal.pone.0236391

**Published:** 2020-07-30

**Authors:** Aan Fleur Terrens, Sze-Ee Soh, Prue Morgan

**Affiliations:** 1 Movement Disorder Program, Peninsula Health, VIC, Australia; 2 Department of Physiotherapy, Monash University, VIC, Australia; 3 Department of Epidemiology and Preventative Medicine, Monash University, VIC, Australia; Universidade Federal do Rio Grande do Sul, BRAZIL

## Abstract

**Background:**

There is growing evidence that aquatic physiotherapy may be effective for people with Parkinson’s Disease (PD) but most studies have investigated land based type exercises in the aquatic environment. Few studies have examined customised aquatic therapies such as the Halliwick concept which focuses on trunk rotation and core stabilisation.

**Objective:**

The primary aim was to determine the feasibility of a Halliwick style aquatic physiotherapy intervention for people with PD. The secondary aim was to compare the Halliwick intervention with traditional aquatic and land based physiotherapy in terms of disease severity, balance and fear of falling.

**Methods:**

Halliwick style aquatic, traditional aquatic and land based physiotherapy were trialled in a single blind pilot study. All interventions ran for 60 minutes per week over 12 weeks. Feasibility outcomes were safety, adherence and attrition. Secondary outcomes included the Unified Parkinson’s Disease Rating Scale motor subsection (UPDRS-III), Berg Balance Scale (BBS), Mini BESTest and modified Falls Efficacy Scale (mFES).

**Results:**

30 participants with moderate PD were recruited. Participant mean age was 72 years (SD 8.4; range 51–86) with moderate disease severity (median Hoehn & Yahr score 3; IQR 1).No falls occurred during intervention sessions, however ten participants reported falls during the study period. No other adverse consequences were reported. All groups had adherence over 85%. No within group significant differences were found in UPDRS-III, BBS or mFES scores post-intervention for all groups, but the Halliwick aquatic group improved significantly in the Mini BESTest post-intervention (p = 0.011, 95% CI -7.36,-1.31, *t* (10) = -2.98).

**Conclusions:**

Despite people with PD being a vulnerable population, aquatic physiotherapy, including the Halliwick style is a safe treatment option. Promising results for balance in the Halliwick aquatic group were observed, but further studies with larger sample sizes is required to increase confidence in the results.

## Introduction

Parkinson’s disease (PD) is a progressive neurological disorder with common motor manifestations such as stooped posture, tremor, bradykinesia, rigidity, festination, freezing of gait and postural instability [1–4]. Postural instability results in a high proportion of falls in people with PD, with studies estimating around 60–70% of people with PD fall at least once a year, and 39–50% have recurrent falls [5–7]. Recent treatment approaches to reduce falls in people with PD include movement strategy training by using attention cues [6], progressive resistance strength training [8], falls education [9], Tai Chi and dancing [6] but the impact of these treatment techniques on postural stability has not been conclusive across the disease spectrum. Recent systematic reviews have indicated that aquatic physiotherapy may be an effective treatment option in this population, and may improve balance and disability outcomes [10–12], however it is not clear what elements of an aquatic physiotherapy program lead to best outcomes. Previous studies have generally focused on traditional aquatic physiotherapy programs that have a mix of aerobic, strengthening and balance exercises [12]. Trunk rigidity in PD may lead to impairment in the vestibular system and therefore postural control [13, 14], and prior research has shown that land based trunk rotational exercises lead to improved balance in other neurological cohorts such as stroke [15]. Little is known about whether specific aquatic therapies such as the Halliwick concept which focuses on complex trunk rotations and core stabilisation may be beneficial for this population.

The principals of aquatic physiotherapy utilise the unique hydrostatic and hydrodynamic properties of water to influence human movement [16]. Aquatic physiotherapy, or hydrotherapy, has been shown to be an effective treatment for individuals post hip and knee joint replacements, those with lower back pain and stroke [16, 17]. Given its successful use in older adults with other conditions, aquatic physiotherapy has the potential to be a useful treatment for adults with PD. Previous research has shown that decreasing axial rigidity, a common symptom in PD, has a positive impact on balance performance [18]. This suggests that the Halliwick style of aquatic therapy may be of value in this population. The Halliwick concept is a 10 step, three stage system that teaches individuals to become completely independent with their movement in the water through different positioning and progressive exercises [19, 20]. The first phase focuses on adjusting to the water environment whilst the second phase teaches individuals to balance and control various types of rotations. Phase two of the Halliwick concept, which has a focus on balance and rotations, may improve axial rigidity, core strength and therefore postural stability in those with PD. Lastly, the third stage uses propulsive movements that are tailored to the individual that allow them to achieve independence in the water environment.

One study has examined the effect of the Halliwick program and found this treatment to significantly improve balance in a small group of people following stroke [21]. A study by Zhu, Yin, Cui, et al. [22] compared parts of the Halliwick concept in a general aquatic program to aquatic obstacle training, but the focus of this study was to reduce freezing of gait. Another study has utilised the Halliwick method with PD patients, but did not use any standardised outcome measures to monitor change in function nor report any safety outcomes [23]. We currently do not know if using the complete 10 step Halliwick concept in people with PD is safe. As there is no previous research using the complete Halliwick concept in people with PD, and there have been no studies that have singularly examined whether it has an impact on balance in this population, further research is warranted to determine whether this concept is a feasible treatment option.

A potential risk associated with aquatic physiotherapy is the challenge to both the cardiovascular and respiratory systems associated with partial or complete immersion in water, potentially amplified when applied to older adults with cardiovascular or respiratory co-morbidities, such as those with PD [24]. Previous research has also shown that safety data is under reported in literature [11]. Therefore, the primary aim of this study was therefore to determine whether aquatic physiotherapy, in particular the complete 10 step Halliwick concept, is a feasible and safe treatment modality for people with PD. The secondary aim was to compare the Halliwick style intervention with traditional aquatic and land based physiotherapy in terms of disease severity, balance and fear of falling.

## Methods

### Design and setting

This study was a single blind pilot study completed between June 2016 and July 2018 in an outpatient public health facility in Melbourne, Australia. Ethics approval was gained from the Peninsula Health Human Research Ethics Committee (HREC) (Project 15/PH/32) and Monash University HREC (Project CF16/1341–2016000731). This study was registered with the Australian New Zealand Clinical Trials Registry (ACTRN12616000834459).

### Participants

Participants were required to have a diagnosis of idiopathic PD confirmed by a neurologist, transfer and walk without assistance with or without gait aid (as participants are required to independently transfer in and out of the pool via steps), and have a Mini Mental State Exam (MMSE) score of 24 or above so that they can follow instructions. Those with unstable medical conditions or a self-reported history of any musculoskeletal, cardiothoracic, other neurological or psychological condition that might potentially affect participation were excluded. If there were any doubt as to medical stability, the participant’s local doctor provided a signed medical assessment form deeming suitability for aquatic and land based physiotherapy. Participants were recruited from local Movement Disorders clinics, private neurologists and from local support groups to ensure the sample would capture the broad characteristics of people with PD. There were no restrictions on disease severity, disease duration or how sedentary participants were. Participants were also sent information regarding the study if they had been involved previously with the Movement Disorders Program at Peninsula Health or if they had telephoned to enquire about the study. A sample size calculation based on a 60% recruitment rate of all participants approached [25], allowing for an attrition rate of 20%, a precision estimate of 20% and 95% confidence interval (CI), resulted in a minimum sample size of 28 participants required for this study. A target sample of 36 was established to aim for equal numbers of participants in the intervention groups.

### Procedure

Participants who expressed interest were screened by telephone, and attended the initial assessment where the blinded assessor confirmed study eligibility and obtained informed consent. Initial testing consisted of cognitive and aquatic safety screening to ensure participants were cognitively and medically suitable for study participation. Block randomisation was used for group allocation, with randomisation order developed by hand, via a third party unrelated to the study. Researchers opened an opaque, sealed and numbered envelope which determined the next intervention group. Following the initial assessment, the participant was given a sealed envelope, prepared by a research assistant, containing their allocated intervention group. There was a maximum of six participants per group, and two cohorts per intervention type. Randomisation determined the order of intervention groups, as only one group ran at a time. Details regarding demographic and clinical characteristics of participants were obtained at baseline testing.

### Interventions

All interventions were delivered by a physiotherapist and allied health assistant experienced in treating people with PD. All interventions were of 60 minutes duration per week for 12 weeks. Pre-intervention testing by the blinded assessor was undertaken one week prior to commencement of the intervention, and follow up post-intervention testing by the blinded assessor occurred one-week after conclusion of the intervention. When participants were on oral medication for their PD, testing and intervention occurred during the”on” stage of their medication cycle.

There were three intervention groups: (1) Halliwick aquatic exercises (core specific exercises and exercises from the Halliwick concept); (2) traditional or current practise aquatic physiotherapy exercises; and (3) land based exercises (control group). Both aquatic interventions took place in a hydrotherapy pool (6m x 10m), with a depth ranging from 1.1m to 1.5m. The water temperature was approximately 34.7 degrees Celsius for all aquatic interventions, with relative humidity ranging from 63% to 76% and pool deck temperature ranging from 25 to 31 degrees Celsius. The characteristics of the exercises delivered in the three intervention groups are described in Table 1, with in depth detail of each intervention shown in S1 Table.

**Table 1:** Intervention characteristics.

	INTERVENTION GROUP		
	*Halliwick Aquatic*	*Traditional Aquatic*	*Land Based*
Walking	✓	✓	✓
Lower Limb	×	✓	✓
Upper Limb	×	✓	✓
Strength	×	✓	✓
Aerobic	×	✓	✓
Balance	✓	✓	✓
Trunk Mobility	✓	✓	✓
Complex Rotations (sagittal, transverse, longitudinal)	✓	×	×
Core Stabilisation	✓	×	×
Stretching	✓	✓	✓

All intervention groups completed walking exercises as a warm up and both upper and lower limb stretching as a cool down. The Halliwick aquatic intervention group completed trunk mobility, core stabilisation and rotational exercises as the primary exercises whereas the other intervention groups completed a range of strength, balance and aerobic exercises. The Halliwick aquatic group followed the Halliwick concept, with participants progressing through each of the 10 steps as able combined with core specific exercises. Most notably, the Halliwick concept contains complex rotational movements where the participant is fully supported by the water, and subsequently require significant core control. The trunk mobility subsection has two standing exercises, where participants’ feet are fixed on the ground. Land based exercises were matched with the traditional aquatic intervention exercises as much as possible in terms of the number of balance and cardiorespiratory exercises, types of stretches and muscle groups targeted. Exercise intensity was measured using the Borg rating of perceived exertion scale [26] during each exercise. Participants were advised to exercise to a level 13–14 on the Borg rating scale, which indicates that they were working somewhat hard in intensity. S1 Table outlines the exercises and progressions in detail, with each participant only progressing if they completed the original exercise independently and safely.

### Outcome measures

Study feasibility was evaluated by examining safety, adherence and attrition. Safety was measured by the absence or presence of adverse events, either participant reported or observed during the intervention sessions. This included falls during the intervention sessions and the intervention period, chlorine allergies, muscle stiffness, pain and fatigue. Adherence was recorded as the number of sessions that each participant attended in the intervention period expressed as a percentage of total sessions offered. If the participant cancelled or did not attend their scheduled session the treating physiotherapist followed up with a phone call, with each reason recorded for every missed session. An adherence rate of more than 70% is deemed acceptable, as this figure has been used in other feasibility studies for people with PD [27]. Attrition was recorded as the number of participants that dropped out of the study during the intervention period. All participants who dropped out were asked to provide a reason if not already given. To be considered satisfactory, we expected an attrition rate of 20%, which is conservative compared to other studies [28, 29] to allow for the fragility of this population.

Secondary outcome measures for disease severity, balance and fear of falling were also recorded. The motor subsection of the Unified Parkinson’s Disease Rating Scale (UPDRS-III) [30] was used to measure the severity and disability associated with the motor manifestations of PD, with higher scores indicating greater level of disability. This section of the UPDRS was developed to measure and rate the different motor manifestations of the disease and has been shown to have good scale reliability and construct validity [31, 32]. Balance was measured using the Berg Balance Scale (BBS) [33] and the Mini BESTest [34]. The BBS was developed to assess balance in the older population, in particular people with PD. The BBS is a reliable and valid measure of balance in this population [35, 36]. The BBS has also been found to have strong correlations with the UPDRS-III, which indicates as disease severity increases there is a correlating decrease in balance [35]. Lower BBS scores indicate reduced balance ability. The Mini BESTest is a valid and reliable predictor of falls in older individuals, including those with PD [36], with lower scores on the scale indicating reduced balance. The modified Falls Efficacy Scale (mFES) [37] assessed how concerned participants were about falling when completing a range of functional tasks. Lower scores on the mFES indicate lower self-confidence and it has been shown to have good construct validity and test-retest reliability within the PD population [38].

### Statistical analysis

All quantitative analyses were completed using SPSS statistical software version 25.0 (SPSS Inc, Chicago, Illinois). Descriptive statistics were used to summarise demographic and clinical characteristics of each group as well as feasibility outcomes of safety, adherence and attrition. Data distribution were visually examined and tested with the Shapiro-Wilk test for normality. To determine whether there was a change in the secondary outcome measures (i.e. disease severity, balance, fear of falling) over the intervention period for each group, paired sample t-tests were used if data were normally distributed. Where data were not normally distributed, Wilcoxon signed-rank tests were used. Differences between groups for secondary outcome measures were explored using one-way analysis of covariance (ANCOVA) to account for potential differences at baseline between intervention groups. A square root transformation of BBS was undertaken to improve the normality and linearity of the residuals. A Bonferroni adjustment was made for post-hoc comparisons between groups to control for the increased risk of Type I errors. There were two main comparisons made in this study; between the Halliwick aquatic and traditional aquatic intervention groups, and between the Halliwick aquatic and land based intervention groups. For an alpha level of 0.05, the *p-*value had to fall below 0.025 to achieve significance. Any missing data from participants who were randomised to the intervention were analysed using intention to treat analysis by using the last data point captured [39].

## Results

### Participants

**Fig 1:**
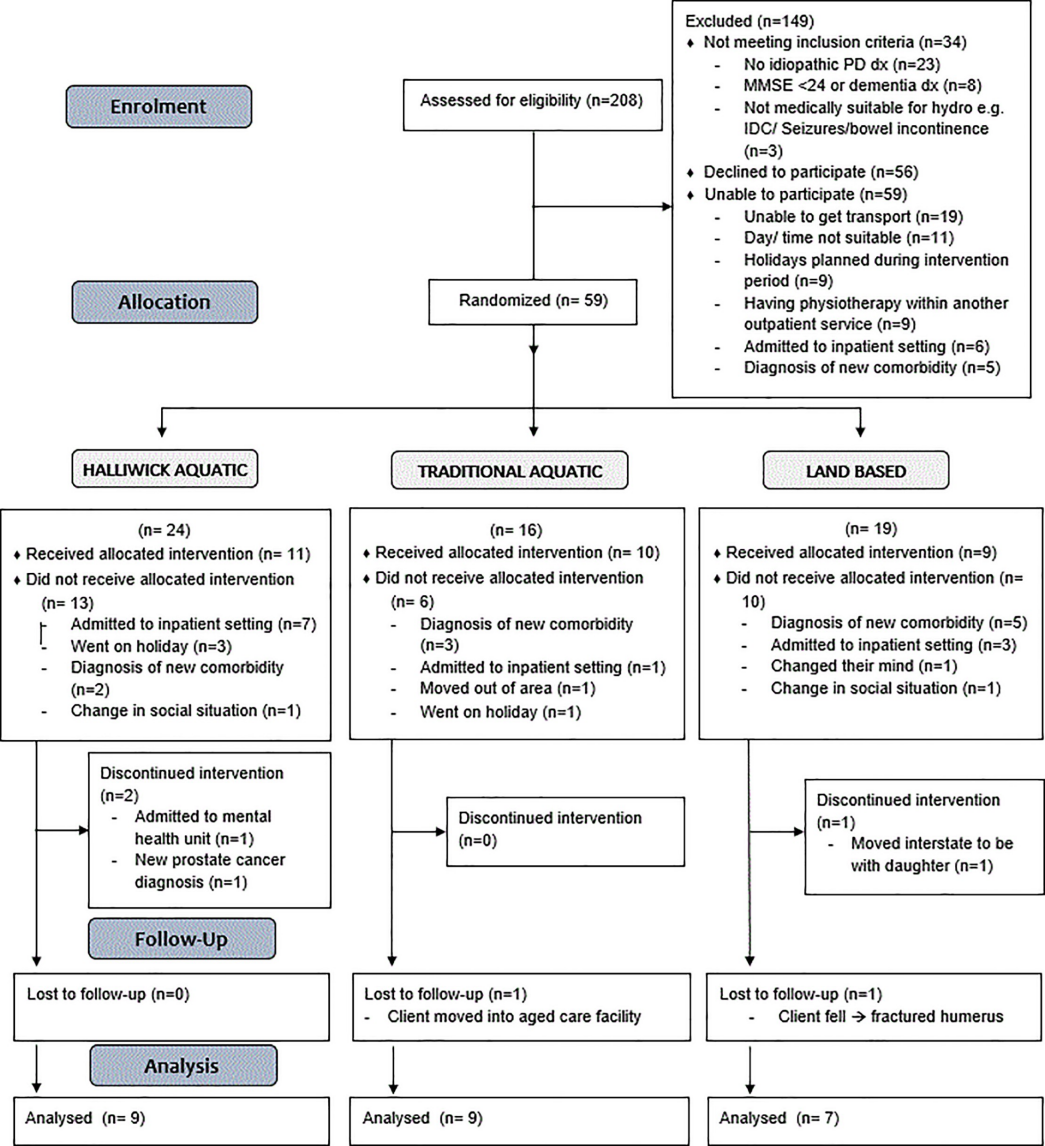
Consort diagram.

A total of 208 individuals with PD were initially assessed for eligibility with 59 individuals randomised: 24 to the Halliwick aquatic intervention, 16 to the traditional aquatic intervention and 19 to the land based intervention. 30 participants (24 males, 6 females) received the allocated intervention (11 Halliwick, 10 traditional, 9 land based) and post intervention measures were obtained from 25 participants (Fig 1). Participants ranged in age from 51 to 86 years (mean 72; SD 8.4) and had moderate disease severity (median Hoehn & Yahr score of 3; IQR 1). 16 participants had fallen once or more in the past 12 months and 21 had a musculoskeletal comorbidity. Participant characteristics are described in Table 2. More males than females were recruited into this study, and this is reflective of the general population of PD [40].

**Table 2:** Characteristics of participants at baseline who received intervention.

	Halliwick Aquatic Intervention Group (n = 11)	Traditional Aquatic Intervention Group (n = 10)	Land Based Intervention Group (n = 9)	All participants (n = 30)
Age, years (mean, SD)	74.1 ± 6.6	65.6 ± 7.7	76.4 ± 7.4	72.0 ± 8.4
Males, n (%)	10 (90)	7 (70)	7 (78)	24 (80)
MMSE (mean, SD)	27.5 ± 2.1	27.6 ± 1.6	27.2 ± 2.1	27.5 ± 1.9
Hoehn & Yahr	3 (1–3)	2 (1–3)	3 (2–3)	3 (1–3)
Disease duration, years (mean, SD)	6.7 ± 6.3	5.2 ± 7.1	4.2 ± 3.1	5.5 ± 5.6
Levodopa use, n (%)				
Yes	11 (100)	9 (90)	7 (78)	27 (90)
No	0	1 (10)	2 (22)	3 (10)
Living situation, n (%)				
Alone	3 (27)	2 (20)	2 (22)	7 (23)
Not alone	8 (73)	8 (80)	7 (78)	23 (77)
Marital status, n (%)				
Single	3 (27)	3 (30)	2 (22)	8 (27)
Married	8 (73)	7 (70)	7 (78)	22 (73)
Co-morbidities^1^, n (%)				
None	1 (9)	1 (10)	0 (0)	2 (7)
Genitourinary	1 (9)	1 (10)	3 (33)	5 (17)
Respiratory	3 (27)	0 (0)	0 (0)	3 (10)
Circulatory	6 (55)	3 (30)	6 (67)	15 (50)
Musculoskeletal	9 (82)	4 (40)	8 (89)	21 (70)
Neoplasms	2 (18)	1 (10)	3 (33)	6 (20)
Mental	2 (18)	2 (20)	3 (33)	7 (23)
Other	3 (27)	6 (60)	4 (44)	13 (43)
Number of falls in past 12 months, n (%)				
None	5 (46)	5 (50)	4 (44)	14 (47)
1 fall:	0	3 (30)	1 (11)	4 (13)
≥2 falls:	6 (55)	2 (20)	4 (44)	12 (40)
UPDRS-III	55 (42.5–60)	46 (41.5–54.8)	37 (32–58)	50.5 (35.5–58.8)
Mini BESTest	22 (14–27)	22 (20.5–28)	18 (18–23)	22 (17.3–27)
Berg Balance Scale	47 (45–51.5)	53 (51.3–54.8)	50 (45–52)	50.5 (45.5–53)
Modified Falls Efficacy Scale	7.6 (4.9–8.5)	6.9 (6.2–8.3)	7.9 (6.8–8.9)	7.1 (5.9–8.6)

All numbers are medians and inter quartile ranges unless otherwise stated.

^1^Classified according to the International Statistical Classification of Diseases and Related Health Problems (ICD-10), MMSE = Mini Mental State Exam, UPDRS-III = Unified Parkinson’s Disease Rating Scale-Part 3

### Primary outcome measures

#### Safety

There were no falls during the intervention sessions for all intervention groups, however, a third of participants (n = 10) reported falls at home during the 12-week intervention period (Table 3). Fallers were equally represented in the Halliwick aquatic and land based intervention groups (n = 4 each) with only two fallers in the traditional aquatic intervention group. There were 32 falls reported across the intervention period. Main reasons for falling were tripping (n = 11), followed by unknown reasons (n = 10), freezing of gait (n = 5), dizziness (n = 4) and legs giving way (n = 2).

**Table 3:** Feasibility data on safety, adherence and attrition on those who received intervention.

	HALLIWICK AQUATIC (n = 11)	TRADITIONAL AQUATIC (n = 10)	LAND BASED (n = 9)	TOTAL (n = 30)
*Falls during 12 week intervention period*, *n (%)* *				
0 falls:	7 (64)	8 (80)	5 (67)	20 (67)
1 fall:	1 (9)	1 (10)	3 (33)	5 (17)
≥2 falls	3 (27)	1 (10)	1 (11)	5 (17)
*Other adverse events*, *n (%)*				
Muscle Pain	0 (0)	1 (10)	2 (22)	3 (10)
Stiffness	1 (9)	2 (20)	2 (22)	5 (17)
Chlorine allergy	0 (0)	0 (0)	n/a	0 (0)
Fatigue	2 (18)	2 (20)	8 (89)	12 (40)
*Adherence*, *%*	90%	85%	91%	89%
*Attrition*, *n (%)*				
Discontinued	2 (18)	0 (0)	1 (11)	3 (10)
Lost to follow up	0 (0)	1 (10)	1 (11)	2 (7)
*BORG*, *mean*	12.2	13.1	16.7	14

*Number of falls reported not within intervention sessions, BORG = BORG scale measuring rate of perceived exertion, averaged over all intervention sessions

Across all intervention groups fatigue was a common side effect post-intervention. This appeared to be more common in participants in the land based intervention group (n = 8). Stiffness (n = 5) and muscle pain (n = 3) were reported by a few participants across all groups. Importantly, no participants from the aquatic groups reported a chlorine allergy (Table 3).

#### Adherence and attrition

Interventions consisted of 12 one-hour weekly classes with an overall adherence rate of 89% (Table 3). Reasons for absences included participant illness, transport issues and planned social events. Of all participants randomised, 54% (n = 13) of individuals in the Halliwick aquatic intervention 38% (n = 6) in the traditional aquatic intervention and 53% (n = 10) in the land based intervention exited the study. Attrition after intervention sessions commenced was due to a variety of reasons including admission to hospital (n = 2) or care facility (n = 1), new medical diagnosis (n = 1) or personal reasons (n = 1).

### Secondary outcome measures

#### Disease severity

**Fig 2:**
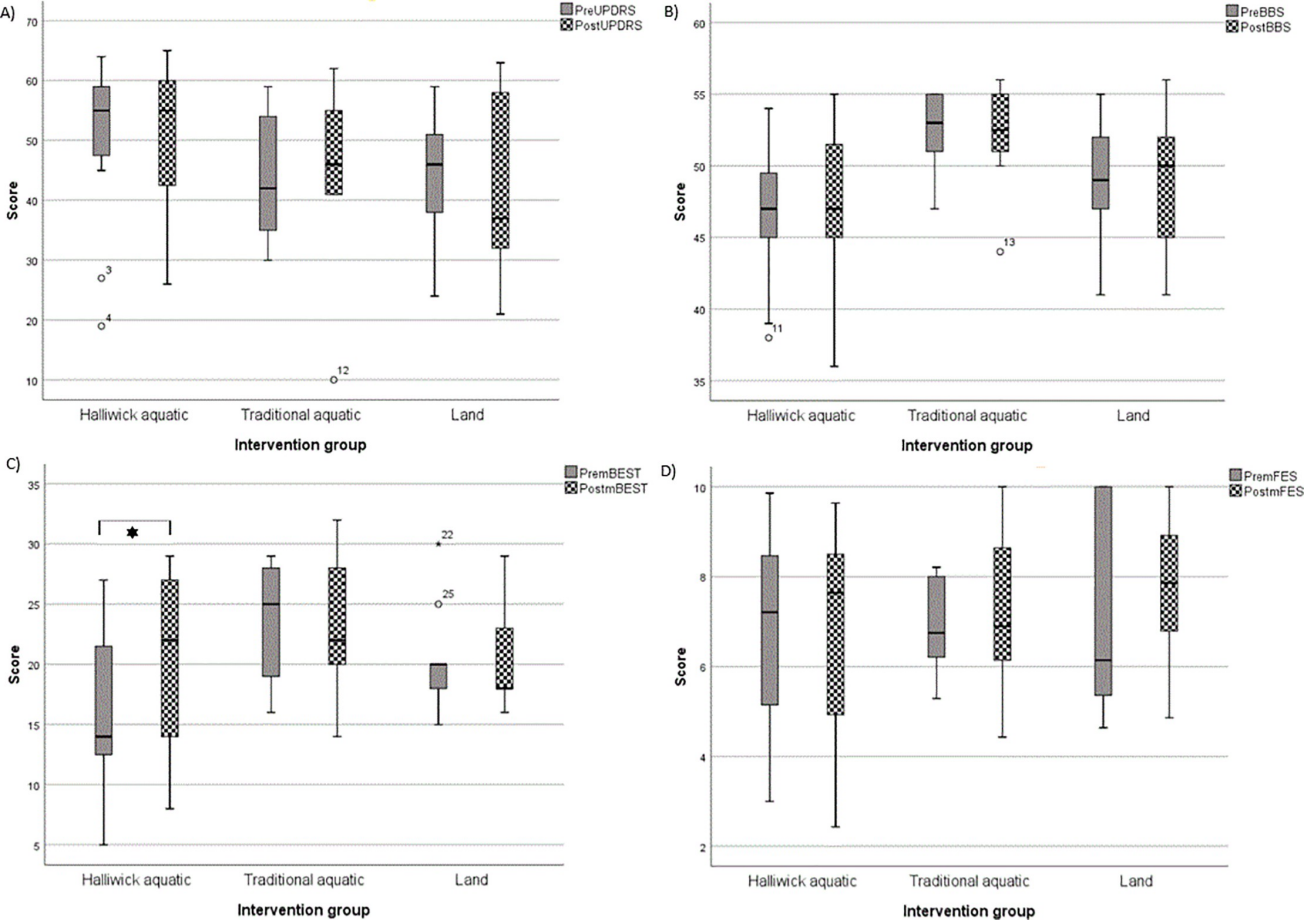
Box plots of outcome measures pre and post intervention for (A) disease severity as measured by the UPDRS-III; (B) balance as measured by the Berg Balance Scale; (C) balance as measured with the Mini BESTest; and (D) fear of falling (mFES). Significant differences between groups are indicated with a star. A box is drawn from the first quartile to the third quartile, with a line passing through the box at the median.

Disease severity, as measured by UPDRS-III scores, improved in the aquatic Halliwick and land based groups post-interventions, however, these were not statistically significant (Fig 2A) (post intervention Halliwick aquatic median 55; IQR 42.5–60; traditional aquatic median 46; IQR 41.5–54.8; land median 37; IQR 32–58). No significant differences were also observed between any intervention groups after adjusting for baseline scores.

#### Balance

There was minimal change in balance when measured using the BBS post intervention in groups after adjusting for baseline scores (Fig 2B) (post intervention Halliwick aquatic median 47; IQR 45–51.5, traditional aquatic median 53; IQR 51.3–54.8, land median 50; IQR 45–52), and no differences were seen between groups post intervention. When balance was measured using the Mini BESTest, a significant improvement was observed in the Halliwick aquatic intervention group (pre-intervention median 14; IQR 12.5–21.5, post-intervention median 22; IQR 14–27) following treatment(p = 0.011, 95% CI -7.36,-1.31, *t* (10) = -2.98) (Fig 2C) (post intervention traditional aquatic median 22; IQR 20.5–28, land median 18; IQR 18–23). No improvements in balance as measured by the Mini BESTest were observed in the other groups. There were also no differences in balance improvement following intervention between the groups when measured using the Mini BESTest after adjusting for baseline scores. It is important to note however, that the Halliwick aquatic intervention group had significantly lower Mini BESTest scores compared to the other intervention groups at baseline (pre intervention Halliwick aquatic median 14; IQR 12.5–21.5, traditional aquatic median 25; IQR 19.8–27.5, land median 20; IQR 18–20). Lower scores on the Mini BESTest indicate worse balance.

#### Fear of falling

There was no significant improvements in fear of falling (as measured by the mFES) post intervention in the Halliwick aquatic intervention group, and there were no differences observed between groups (Fig 2D).

## Discussion

The primary aim of this study was to determine whether a Halliwick aquatic physiotherapy intervention that focuses on trunk rotation and core stabilisation (including the Halliwick concept) is a feasible and safe treatment modality in people with PD. Our results indicate that aquatic physiotherapy, including the Halliwick style, is a safe treatment option in this population. Minimal side effects for all participants were observed, and participants in the land based group appeared to report more fatigue compared to those in the aquatic intervention groups. This suggests that the hydrostatic and hydrodynamic properties of the aquatic environment may provide support for joints and aid in muscle relaxation for people with PD [24]. Additionally, none of the participants in the two aquatic intervention groups reported side effects such as chlorine allergies or skin rashes, which is consistent with previous studies [11]. Despite this population being more susceptible to orthostatic hypotension [41], no cardiovascular issues were observed during the trial. Thus, from a safety perspective, aquatic physiotherapy including the Halliwick concept, is just as safe as land based therapy for people with PD.

Although there were no falls observed during sessions, participants in all groups reported a high number of falls (n = 32) throughout the intervention period. Of the 30 participants in this study, 10 reported falls during the 12 week intervention period, with a higher proportion of individuals falling more than twice in the Halliwick aquatic intervention group (n = 3). Amongst the five participants who were multiple fallers, there were 27 falls reported, confirming that people with PD are at a high risk of falls and are a very vulnerable population [7]. This high falls risk needs to be taken into consideration when treating people with PD whether in a land based or aquatic environment.

Whilst adherence to therapy was good, with over 85% adherence for all intervention groups, there was a low uptake of participants into this study. We contacted 208 individuals and although one quarter were not eligible, the majority declined to participate or were unable to attend. The main reason for declining to participate was that participants were not interested in water exercises (n = 36), with the main reasons for being unable to participate being transport (n = 19) and availability (n = 11) difficulties. The proportion of males and females that declined to participate or were unable to participate were reflective of the PD population, with a larger proportion of males compared to females [40, 42]. In addition, after being accepted into the study, another 29 participants were unable to participate. Our recruitment experience indicate that there may be several barriers towards participating in exercise therapy amongst people with PD. This is consistent with existing literature which reports low participation rates in older people for falls prevention programs or home exercise programs [43, 44]. The large number of drop outs, combined with the number of participants unable to commence therapy due to changes in health status highlights the medical fragility of this population, and therefore strategies are required to optimise engagement with this vulnerable population.

Although this study was not powered to show differences in disease severity and balance outcomes, we observed a significant improvement in Mini BESTest scores for the Halliwick aquatic intervention group following intervention. It should be noted that the Halliwick aquatic intervention group had significantly different MiniBESTest scores at baseline although this was adjusted for in the analyses. Recent systematic reviews have shown that aquatic physiotherapy in PD results in improved balance (as measured by the BBS) when compared to land based physiotherapy [10, 45], but it is not clear what elements of aquatic intervention lead to these improvements. Future studies may wish to focus on determining if aquatic physiotherapy broadly results in improvements in balance and fear of falling, or whether specific components of an aquatic program are more effective than others.

### Limitations

A number of limitations need to be noted. Firstly, this study was not designed to determine the efficacy of aquatic physiotherapy including the Halliwick concept for the secondary outcome measures and an adequately powered study is warranted to explore potential effects on outcomes including balance, fear of falling or disease severity. For instance, in order to confirm whether the Halliwick aquatic intervention program was more effective compared to traditional aquatic programs, a sample size of 56 will be required in each group to detect a clinically significant difference of 5.52 points [36] in balance as measured by the MiniBESTest with 80% power and alpha set at 5%. Another limitation to note is that exercise intensity was only measured using the BORG scale, which is a self-reported measure of perceived exertion, and therefore may not be an accurate measure as participants may under or overestimate their level of exertion. We also acknowledge that care needs to be taken when generalising study findings given the small sample size and that we only included participants with mild to moderate PD. Nevertheless, this is the first study to examine whether a Halliwick aquatic therapy approach can be used safely in this population. We have ensured that all safety criteria relating to aquatic physiotherapy in PD were reported and used a minimum dataset that includes the UPDRS-III allow for accurate study evaluation and replication [11]. As the focus of this study was on feasibility, and in particular safety, we did not follow the European Physiotherapy Guidelines for Parkinson’s Disease [46] in terms of exercise prescription. This may explain why no significant effects were observed in the secondary outcome measures. It is recommended that future studies meet these training guidelines to determine treatment efficacy.

## Conclusions

People with PD are a vulnerable population and are at high risk of falls. Aquatic physiotherapy, including a novel approach such as the Halliwick concept, is a feasible and safe treatment option for people with PD and may have a positive impact on balance and fear of falling. However, a larger scale study is required to explore this further.

## Supporting information

S1 Checklist CONSORT 2010 checklist of information to include when reporting a randomized trial^a^. (PDF)

S1 Table Details of interventions. BOS: base of support, UL: upper limb.
(DOCX)

S1 Protocol (DOC)
