# Supplementary material for: The safety and feasibility of a Halliwick style of aquatic physiotherapy for falls and balance dysfunction in people with Parkinson's Disease: A single blind pilot trial
Source: PLoS One. 2020 Jul 30;15(7):e0236391. doi: 10.1371/journal.pone.0236391 (PMC7392279; doi:10.1371/journal.pone.0236391)
Supplement: S1 Table — BOS: base of support, UL: upper limb. (DOCX) [file pone.0236391.s002.docx]

Table 1. Details of interventions

| **HALLIWICK AQUATIC** | | | | | |
| --- | --- | --- | --- | --- | --- |
| **Exercise** | **Depth** | **Exercise description** | **Time** | **Progression** |  |
| **Warm up:**   1. Walking Forwards 2. Backwards 3. Sideways | Xiphisternum  Xiphisternum  Xiphisternum | Walking laps across pool, emphasis on big steps/ arm swing. | 5 mins for all |  |  |
| **Halliwick Concept Movements:**   1. Breath control 2. Floating 3. Upthrust jumps | 1.2m  1.2m  1.2m | 1. Prone, hanging onto the rail 2. Supine, floating on the water 3. Bringing feet off ground to feel water supporting them | 10 mins for all | 1. Not holding onto rail 2. Adding turbulence, adding arms for swimming 3. Closing eyes |  |
| **Halliwick Concept Rotations:**   1. Dumbell glides to L) and R) 2. Supine to box sit 3. Supine to prone 4. Longitudinal rolls to L) and R) | 1.5m  1.2m  1.2m  1.2m | 1. Standing with arms out by side, gliding to L) and R) 2. Therapist assisted close to rail, with dumbells 3. Therapist assisted close to rail, with dumbells 4. Therapist assisted | 5 mins  5 mins  5 mins  5 mins | 1. No dumbells 2. No dumbells, verbal cues only, then no verbal cues. Away from rail with no therapist support 3. No dumbells, then supine-prone-supine rolls. Away from rail with no therapist support 4. No assistance, then combined rotations (ie swimming) |  |
| **Core stabilisation/ Trunk mobility/ Balance:**   1. Trunk Rotation 2. Prone Kicking 3. Trunk Extension 4. Reaching outside BOS forwards/ backwards/ L) and R) | 1.2m  1.2m  1.2m  1.2m | 1. Noodle trunk rotation to L) and R) 2. Holding onto rail, focus on core stabilisation not moving legs 3. Holding onto the rail behind, feet touching wall, stretching forwards 4. Reaching outside BOS, shifting weight onto toes (fwds)/ heels (bwds), without losing balance, holding onto dumbells | 5 mins  5 mins  5 mins  5 mins | 1. Resistance with paddles 2. Addition of flippers 3. Nil 4. No dumbells |  |
| **Cool down:**   1. Stretches | 1.2m | 1. Gentle calf stretches on step/ Hamstring stretch/ Quadriceps stretch/ Triceps and Biceps stretches | 5 mins |  |  |
| **TRADITIONAL AQUATIC** | | | | | |
| **Exercise** | **Depth** | **Exercise description** | **Time** | **Progression** |  |
| **Warm up:**   1. Walking Forwards 2. Backwards 3. Sideways | Xiphisternum  Xiphisternum  Xiphisternum | Walking laps across pool, emphasis on big steps/ arm swing. | 5 mins for all |  |  |
| **Aerobic:**   1. Cycling 2. Step ups/ downs | 1.2m  1.2m | 1. Cycling in the corner of the pool, holding onto rails, neck and bottom floats if required 2. Stepping up/ down on step in the water, nil UL support | 5 mins  5 mins | 1. Nil 2. Jumping over step |  |
| **Trunk mobility:**   1. Trunk Rotation 2. Lateral flexion stretch | 1.5m  1.2m | 1. Feet fixed, noodle trunk rotation to L) and R) 2. Arms down by side, reaching down towards knees without going into trunk flexion | 5 mins   1. mins | 1. Resistance with paddles 2. Nil |  |
| **Balance:**   1. Single leg Stance 2. Kickboard pushdowns | 1.2m  1.2m | 1. Balancing on one leg, no UL support 2. Kickboard under one foot, balancing on one leg, pushing board up and down. Alternating L) and R) | 5 mins  5 mins | 1. Eyes closed 2. Thicker kickboard |  |
| **Lower limb strength:**   1. Single leg calf raises/ squats 2. Flipper Kicking | 1.2m  1.2m | 1. Holding onto rail, gentle calf raises and squats on one leg. Alternating L) and R) 2. Sitting on plinth, knee flexion and extension whilst kicking | 5 mins  5 mins | 1. No UL support 2. Nil |  |
| **Upper limb strength:**   1. Hydrotones | 1.5m | 1. Punches forwards/ lateral flexion/ shoulder flexion extension/ Bicep curls | 5 mins | 1. Nil |  |
| **Cool down:**   1. Walking forwards 2. Stretches | Xiphisternum  1.2m | 1. Walking laps across pool, emphasis on big steps/ arm swing. 2. Gentle calf stretches on step/ Hamstring stretch/ Quadriceps stretch/ Triceps and Biceps stretches | 5 mins  5 mins |  |  |
| **LAND BASED** | | | | | |
| **Exercise** | **Depth- N/A** | **Exercise description** | **Time** | **Progression** |  |
| **Warm up:**   1. Walking Forwards 2. Backwards 3. Sideways |  | Walking laps in rails, emphasis on big steps. | 5 mins for all |  |  |
| **Aerobic:**   1. Bike Riding 2. Stepper |  | 1. Seated bike at challenging pace (determined by client) 2. Mini stepper machine, client to do as many steps as possible within time | 5 mins  5 mins | 1. Increase speed 2. Nil |  |
| **Trunk mobility:**   1. Trunk Rotation 2. Lateral flexion stretch |  | 1. Standing trunk rotation in rails 2. Standing arms down by side, reaching down towards knees without going into trunk flexion | 5 mins  5 mins | 1. Nil 2. Nil |  |
| **Balance:**   1. Rockerboard 2. Single leg stance |  | 1. Rockerboard, weight shifting from side to side, and forwards/ backwards 2. Balancing on one leg in rails | 5 mins  5 mins | 1. Nil 2. Cone Tapping in a semi-circle whilst SLS |  |
| **Lower limb strength:**   1. Single leg calf raises/ squats 2. Sit to Stand |  | 1. Holding onto rail, gentle calf raises and squats on one leg. Alternating L) and R) 2. Standing up and sitting down from 45cm chair, nil UL support | 5 mins  5 mins | 1. No UL support 2. Addition of 5kg weighted vest |  |
| **Upper limb strength:**   1. Scapular retraction |  | 1. Scapular retraction with theraband | 5 mins | 1. Increase theraband resistance |  |
| **Cool down:**   1. Treadmill 2. Stretches |  | 1. Walking on treadmill, hands on 2. Gentle calf stretches on step and sitting/ Hamstring stretch/ Quadriceps stretch/ Triceps and Biceps stretches | 5 mins  5 mins |  |  |

BOS: base of support, UL: upper limb
