# Supplementary material for: The safety and feasibility of a Halliwick style of aquatic physiotherapy for falls and balance dysfunction in people with Parkinson's Disease: A single blind pilot trial
Source: PLoS One. 2020 Jul 30;15(7):e0236391. doi: 10.1371/journal.pone.0236391 (PMC7392279; doi:10.1371/journal.pone.0236391)
Supplement: S1 Protocol — (DOC) [file pone.0236391.s003.doc]

| **1.** | **Front Page *** |
| --- | --- |

HREC/15/PH/32.

The effects on balance and falls in those with Parkinson's Disease: A comparison between land based, traditional aquatic and novel aquatic physiotherapy

**3, 02/11/2015**

|  | **STATEMENT OF COMPLIANCE** |
| --- | --- |
|  | This document is a protocol for a clinical research study. The study will be conducted in compliance with all stipulations of this protocol, the conditions of ethics committee approval, the NHMRC National Statement on Ethical Conduct in Human Research (2007) and (if applicable) the Note for Guidance on Good Clinical Practice (CPMP/ICH-135/95). |

|  | **Principal Investigator:** |
| --- | --- |
|  | Fleur Terrens |
|  | **Associate Investigator(s):** |
|  | Prue Morgan  Wendy Toogood |

| **2.** | **Synopsis (250 – 300 words)** |
| --- | --- |
|  | Aquatic Physiotherapy and Land based Physiotherapy is currently used in the management of Parkinson’s disease within the Movement Disorders Program at Peninsula Health. Land based physiotherapy has shown to have a positive effect on gait and balance parameters in those with Parkinson’s Disease, however aquatic physiotherapy has had little research. Further understanding of exercise and movement in this environment is warranted to see if either therapy is more effective at improving balance and gait measures in those with Parkinson’s Disease. |

| **3.** | **Introduction / Background *** |
| --- | --- |
|  | Movement disorders are syndromes where the body’s normal movement patterns are changed. There can be too much movement or not enough. Parkinson’s disease is the most common movement disorder, affecting more than 50,000 people in Australia. It is a progressive neurological condition.  Falls occur in around 70% of people with Parkinson’s Disease (PWP), the risk being around twice that of other community dwellers (Wood, Bilclough, Bowron & Walker, 2002). Balash, Peretz, Leibovich, Herman, Hausdorff and Giladi (2005) found that around 33% of PWP can be classified as fallers, having had 2 or more falls in the past 12 months. Falls due to postural instability can lead to significant morbidity and mortality, with around 65% of falls causing an injury (Wielinski, Erickson-Davis, Wichmann, Walde-Douglas & Pasashos, 2005).  As a high proportion of PWP fall and the likelihood of injury is high, physiotherapy treatment for PD has been directed towards improving balance. For many years treatment was land based only, focussing on movement strategy training for patient’s motor symptoms (i.e. freezing) with some studies investigating the impact of strength training. Currently, only a small proportion of studies have investigated the impact of aquatic physiotherapy, or hydrotherapy on function in PWP.  A systematic review by Geytenbeek (2002) found that there is moderate to high quality evidence to support aquatic physiotherapy in a wide range of conditions, with Montagne, Santos, Battistuzzo and Loureiro (2014) concluding that using the Halliwick method in aquatic physiotherapy was a useful tool that improved balance in stroke survivors. There has been one study that has compared land based therapy and aquatic physiotherapy in PWP, revealing that there were greater gains in balance measures for the aquatic physiotherapy group (Vivas, Arias & Cudeiro, 2011). A further study compared low intensity exercises and muscular resistance exercises in aquatic physiotherapy, and found that muscular resistance exercises resulted in improvements in functional mobility in PWP (Ayan and Cancela, 2012). Zotz, Souza, Israel and Loureiro (2013) reported findings of a study using the Halliwick aquatic physiotherapy method in PWP, but it only compared progress through the Halliwick method, thus not providing any information on how the patient’s balance and mobility changed on land.  Evidence to date does not confirm how best to use aquatic physiotherapy to treat PWP that will reduce falls. My proposed study aims to add to the knowledge regarding the efficacy of this treatment modality to optimise balance and subsequently decrease falls in PWP. |

| **4.** | **Objectives *** |
| --- | --- |
|  | The aim of this research is to examine whether practising transverse, sagittal and longitudinal rotations as described in the Halliwick aquatic physiotherapy model impacts on postural control and balance on land in PWP. We want to compare balance and quality of life test results for this type of aquatic physiotherapy to traditional aquatic physiotherapy exercises and land based exercises, to see if any particular type of exercise improves balance more than other types of exercise.  As there is a limited number of studies in this area of physiotherapy, this research can guide future treatment options. |

| **5.** | **Personnel** |
| --- | --- |

| **5.1** | **Principal Investigator** | | | | | |
| --- | --- | --- | --- | --- | --- | --- |
|  | **Title** | Miss | **First Name** | Aan (Fleur) | **Surname** | Terrens |
|  | **Organisation** | Peninsula Health | | **Department** | Physiotherapy | |
|  | **Appointment** | Physiotherapist | | **Qualifications** | Honours degree of Bachelor of Physiotherapy | |
|  | **Email** | fterrens@phcn.vic.gov.au | | | | |

| **5.2** | **Associate Investigator(s)** | | | | | |
| --- | --- | --- | --- | --- | --- | --- |
|  | **Title** | Associate Professor | **First Name** | Prue | **Surname** | Morgan |
|  | **Mailing Address** | Physiotherapy department, Monash University Peninsula Campus | | | | |
|  | **Suburb/City** | Frankston | | **Postcode** | 3199 | |
|  | **Organisation** | Monash University | | **Dept** | Physiotherapy | |
|  | **Appointment** | Head of Physiotherapy | | **Qualifications** | DOCTOR OF PHILOSOPHY  Year awarded: 2014  MASTER OF APPLIED SCIENCE  Year awarded: 2000  GRADUATE DIPLOMA IN NEUROSCIENCES  Year awarded: 1992  BACHELOR OF APPLIED SCIENCE IN PHYSIOTHERAPY  Year awarded: 1984 | |

| **Title** | Ms | **First Name** | Wendy | **Surname** | Toogood |
| --- | --- | --- | --- | --- | --- |
| **Organisation** | Peninsula Health | | **Dept** | Physiotherapy | |
| **Appointment** | Allied Health Assistant | | **Qualifications** | TC1 AHA | |

| **6.** | **Study Design *** |
| --- | --- |
| 6.1 | Study Description * |
|  | Randomised controlled trial looking at differences in balance and quality of life measures between traditional aquatic physiotherapy exercises and novel rotational practise. This will be compared to land based exercises. |

| 6.2 | Study comparison and interventions * |
| --- | --- |
|  | Participants will be required to attend once weekly for 1 hour, for a total of 12 weeks. Participants will have the option to attend a focus group.  Patients in both aquatic and the land based physiotherapy groups will receive a 60 minute exercise class.  INTERVENTIONS   1. Traditional Aquatic Physiotherapy group  | **Exercise** | **Depth** | **Exercise description** | **Time** | **Progression** | | --- | --- | --- | --- | --- | | **Warm up:**   1. Walking Forwards 2. Backwards 3. Sideways | Xiphisternum  Xiphisternum  Xiphisternum | Walking laps across pool, emphasis on big steps/ arm swing. | 5 mins for all |  | | **Aerobic:**   1. Cycling 2. Step ups/ downs | 1.2m  1.2m | 1. Cycling in the corner of the pool, holding onto rails, neck and bottom floats if required 2. Stepping up/ down on step in the water, nil UL support | 5 mins  5 mins | 1. Nil 2. Jumping over step | | **Trunk mobility:**   1. Trunk Rotation 2. Lateral flexion stretch | 1.5m  1.2m | 1. Feet fixed, noodle trunk rotation to L) and R) 2. Arms down by side, reaching down towards knees without going into trunk flexion | 5 mins   1. mins | 1. Resistance with paddles 2. Nil | | **Balance:**   1. Single leg Stance 2. Kickboard pushdowns | 1.2m  1.2m | 1. Balancing on one leg, no UL support 2. Kickboard under one foot, balancing on one leg, pushing board up and down. Alternating L) and R) | 5 mins  5 mins | 1. Eyes closed 2. Thicker kickboard | | **Lower limb strength:**   1. Single leg calf raises/ squats 2. Flipper Kicking | 1.2m  1.2m | 1. Holding onto rail, gentle calf raises and squats on one leg. Alternating L) and R) 2. Sitting on plinth, knee flexion and extension whilst kicking | 5 mins  5 mins | 1. No UL support 2. Nil | | **Upper limb strength:**   1. Hydrotones | 1.5m | 1. Punches forwards/ lateral flexion/ shoulder flexion extension/ Bicep curls | 5 mins | 1. Nil | | **Cool down:**   1. Walking forwards 2. Stretches | Xiphisternum  1.2m | 1. Walking laps across pool, emphasis on big steps/ arm swing. 2. Gentle calf stretches on step/ Hamstring stretch/ Quadriceps stretch/ Triceps and Biceps stretches | 5 mins  5 mins |  |   B. Novel Aquatic Physiotherapy group   | **Exercise** | **Depth** | **Exercise description** | **Time** | **Progression** | | --- | --- | --- | --- | --- | | **Warm up:**   1. Walking Forwards 2. Backwards 3. Sideways | Xiphisternum  Xiphisternum  Xiphisternum | Walking laps across pool, emphasis on big steps/ arm swing. | 5 mins for all |  | | **Halliwick Concept Movements:**   1. Breath control 2. Floating 3. Upthrust jumps | 1.2m  1.2m  1.2m | 1. Prone, hanging onto the rail 2. Supine, floating on the water 3. Bringing feet off ground to feel water supporting them | 10 mins for all | 1. Not holding onto rail 2. Adding turbulence, adding arms for swimming 3. Closing eyes | | **Halliwick Concept Rotations:**   1. Dumbell glides to L) and R) 2. Supine to box sit 3. Supine to prone 4. Longitudinal rolls to L) and R) | 1.5m  1.2m  1.2m  1.2m | 1. Standing with arms out by side, gliding to L) and R) 2. Therapist assisted close to rail, with dumbells 3. Therapist assisted close to rail, with dumbells 4. Therapist assisted | 5 mins  5 mins  5 mins  5 mins | 1. No dumbells 2. No dumbells, verbal cues only, then no verbal cues. Away from rail with no therapist support 3. No dumbells, then supine-prone-supine rolls. Away from rail with no therapist support 4. No assistance, then combined rotations (ie swimming) | |  |  |  |  | 1. Resistance with paddles 2. Addition of flippers 3. Nil 4. No dumbells |   C. Land Based Physiotherapy group   | **Exercise** | **Depth- N/A** | **Exercise description** | **Time** | **Progression** | | --- | --- | --- | --- | --- | | **Warm up:**   1. Walking Forwards 2. Backwards 3. Sideways |  | Walking laps in rails, emphasis on big steps. | 5 mins for all |  | | **Aerobic:**   1. Bike Riding 2. Stepper |  | 1. Seated bike at challenging pace (determined by client) 2. Mini stepper machine, client to do as many steps as possible within time | 5 mins  5 mins | 1. Increase speed 2. Nil | | **Trunk mobility:**   1. Trunk Rotation 2. Lateral flexion stretch |  | 1. Standing trunk rotation in rails 2. Standing arms down by side, reaching down towards knees without going into trunk flexion | 5 mins  5 mins | 1. Nil 2. Nil | | **Balance:**   1. Rockerboard 2. Single leg stance |  | 1. Rockerboard, weight shifting from side to side, and forwards/ backwards 2. Balancing on one leg in rails | 5 mins  5 mins | 1. Nil 2. Cone Tapping in a semi-circle whilst SLS | | **Lower limb strength:**   1. Single leg calf raises/ squats 2. Sit to Stand |  | 1. Holding onto rail, gentle calf raises and squats on one leg. Alternating L) and R) 2. Standing up and sitting down from 45cm chair, nil UL support | 5 mins  5 mins | 1. No UL support 2. Addition of 5kg weighted vest | | **Upper limb strength:**   1. Scapular retraction |  | 1. Scapular retraction with theraband | 5 mins | 1. Increase theraband resistance | | **Cool down:**   1. Treadmill 2. Stretches |  | 1. Walking on treadmill, hands on 2. Gentle calf stretches on step and sitting/ Hamstring stretch/ Quadriceps stretch/ Triceps and Biceps stretches | 5 mins  5 mins |  | |
|  |  |

| 6.3 | Participants * |
| --- | --- |
|  | A total of 36 participants (12 in each group) with a confirmed diagnosis of Parkinson’s Disease will be admitted into the program, with no self-reported history of any musculoskeletal, cardiothoracic, other neurological or psychological condition that might potentially affect their therapy. Patients must be medically stable to participate. If there is any doubt to medical stability, the patient’s GP must sign a medical assessment form deeming the patient suitable for hydrotherapy and land based physiotherapy.  Patients must not be currently attending the aquatic or land physiotherapy groups at Peninsula Health and must be happy to participate in any treatment group as allocation is randomised.  Patients must have be able to walk unaided +/- gait aid, have an MMSE (mini mental state exam) score of more than 24 to show sufficient cognition for informed consent. If recruitment targets are not met by the end of the timeline, recruitment will cease and analysis will be performed on the participants that have been recruited. |

| 6.4 | Study procedure * |
| --- | --- |
|  | Recruitment through the Movement Disorder Program at Peninsula Health, combined with contacting local support groups and neurologists should attract the desired number of participants.  Participants that show interest will have the PICF and recruitment letter sent out to them, followed by a phone call 2 weeks later.  Participants that are interested in taking part will attend a screen with a blind assessor where they will be assessed for medical suitability for hydrotherapy and land based physiotherapy, and cognition testing, and where demographic and baseline data is collected. If they are suitable for the study, they will then be randomly allocated into either the land based, traditional aquatic or novel aquatic physiotherapy groups.  All participants will attend for one hour weekly for 12 weeks. At the end of the 12 weeks, participants will be blindly re-assessed and asked to complete a patient satisfaction survey, and then will be offered to attend a focus group to discuss the therapy. |

| 6.5 | Outcome(s) * |
| --- | --- |
|  | - Parkinson’s Disease Questionnaire 39 - Personal Wellbeing Index-Adult - Berg Balance Scale - Mini BEST test - UPDRS part 3- motor subscale - Feasibility – Attrition, Adherence, Adverse events - Falls Efficacy Scale |

| 6.6 | Data Collection * |
| --- | --- |
|  | As per usual practice MDP- through Physiotherapy Assessment and Re-Assessment |

| 6.7 | Expected Duration of trial and start times (include a timeline if possible) |
| --- | --- |
|  | March-Dec 2016 – Recruitment of participants  June-Dec 2016 - complete the initial assessments  July 2016 to June 2018 – commence and complete therapy in 3 month blocks, 6 participants in each treatment group, perform 3 month reviews and focus group  June to Dec 2018- Collate results and perform data analyses  Timeframes may alter to earlier dates depending on recruitment progress. These timeframes are allowing for poor recruitment rates. |

| 6.8 | Participant withdrawal |
| --- | --- |
|  | Patients may decline to be involved in the research and this will not affect any current or future treatment through CRP. |

| **7.** | **Data Management *** |
| --- | --- |
|  | Data will be de-identified in a password protected folder on the Peninsula Health hard drive as it is routinely backed up.  Data will be kept for 5 years to assist with Masters of Phil write up, as per Australian code for the responsible conduct of research.  Data will be re-identifiable.  Codes that identify participants will only be accessible to the Principal Investigator. |

| **8.** | **Statistical considerations / Planned Analysis *** |
| --- | --- |
|  | Analysis will be performed through the RevMan/ SPSS statistical packages. A range of descriptive statistics will be applied to data to describe the participants. Additional parametric and non-parametric statistical methods will be used to explore the differences between groups.  Assistance with data analysis will be provided through my masters of phil supervisors Associate Prof Prue Morgan and Dr Sze-Ee Soh. Monash University is also able to provide support with analysis and provision of the statistical packages. |

| **9.** | **Quality Assurance, monitoring and safety** |
| --- | --- |
|  | Peninsula Health CPG’s/ OPG’s   - Clinical Practice Guideline Eligibility, Referral and Admission - Aquatic Physiotherapy screening - Aquatic Physiotherapy Safety and Pool Rescue - Physiotherapy Basic Life Support   Any adverse events (none predicted due to study being usual practise) will be reported to seniors and this board. Medical assistance will be sought if required, and patients will be withdrawn from the project.  All exercises undertaken in the pool will be with a physiotherapist, with an additional allied health assistant pool side as per current physiotherapy safety requirements. |

| **10.** | **Ethical issues** |
| --- | --- |
|  | There are no ethical issues. Assessment and treatment will relate to usual Physiotherapy care. Any participants not wishing to be part of the research or withdrawing will still receive physiotherapy as per usual practise. |

| **11.** | **Finance and resource use *** |
| --- | --- |
|  | As per current Physiotherapy staffing allocation to the Movement Disorders Program. |

| **12.** | **Publication / Authorship** |
| --- | --- |
|  | As per the Principal and associate investigators |

| **13.** | **Limitations of the study & future directions** |
| --- | --- |
|  | This is a functional collection of data and not a complex randomised trial. There will be bias in the treatment programs as there will not be blinding, and this study will only be looking at a small sample size. |
